# Role of Envelope Glycoprotein Complexes in Cell-Associated Spread of Human Cytomegalovirus

**DOI:** 10.3390/v13040614

**Published:** 2021-04-02

**Authors:** Nina Weiler, Caroline Paal, Kerstin Adams, Christopher Calcaterra, Dina Fischer, Richard James Stanton, Dagmar Stöhr, Kerstin Laib Sampaio, Christian Sinzger

**Affiliations:** 1 Institute for Virology, Ulm University Medical Center, 89089 Ulm, Germany; nina.weiler@uni-ulm.de (N.W.); caroline.paal@uniklinik-ulm.de (C.P.); ker-ad@gmx.de (K.A.); christopher.calcaterra@uni-ulm.de (C.C.); dinafischer@gmx.net (D.F.); dagmar.stoehr@uniklinik-ulm.de (D.S.); kerstin.laib@uni-ulm.de (K.L.S.); 2 Division of Infection and Immunity, Cardiff University School of Medicine, Cardiff CF14 4XN, UK; stantonrj@cardiff.ac.uk

**Keywords:** cytomegalovirus, clinical isolates, cell-associated spread, glycoproteins, trimer, pentamer

## Abstract

The role of viral envelope glycoproteins, particularly the accessory proteins of trimeric and pentameric gH/gL-complexes, in cell-associated spread of human cytomegalovirus (HCMV) is unclear. We aimed to investigate their contribution in the context of HCMV variants that grow in a strictly cell-associated manner. In the genome of Merlin pAL1502, the glycoproteins gB, gH, gL, gM, and gN were deleted by introducing stop codons, and the mutants were analyzed for viral growth. Merlin and recent HCMV isolates were compared by quantitative immunoblotting for expression of accessory proteins of the trimeric and pentameric gH/gL-complexes, gO and pUL128. Isolates were treated with siRNAs against gO and pUL128 and analyzed regarding focal growth and release of infectious virus. All five tested glycoproteins were essential for growth of Merlin pAL1502. Compared with this model virus, higher gO levels were measured in recent isolates of HCMV, and its knockdown decreased viral growth. Knockdown of pUL128 abrogated the strict cell-association and led to release of infectivity, which allowed cell-free transfer to epithelial cells where the virus grew again strictly cell-associated. We conclude that both trimer and pentamer contribute to cell-associated spread of recent clinical HCMV isolates and downregulation of pentamer can release infectious virus into the supernatant.

## 1. Introduction

Human cytomegalovirus (HCMV) is a herpesvirus that is prevalent in 45–100% of the population worldwide and causes significant morbidity under conditions of reduced immune defenses [1]. In immunocompetent hosts, a usually subclinical primary infection is followed by a lifelong latency from which the virus can reactivate despite robust cellular and humoral immune responses. While numerous viral modulators of antigen presentation allow escape from virus-specific cytotoxic T cells [2], the ability for cell-associated spread is considered a viral means to evade neutralizing antibodies [3,4,5,6]. After reactivation, the virus can be shed in various body fluids as cell-free infectivity [7].

Animal models with murine cytomegalovirus (MCMV) suggest that the cell-free mode of transmission may be particularly relevant for host-to-host transmission, whereas within-host dissemination can rely on the cell-associated mode of viral spread [8,9]. For HCMV, this assumption is supported by clinical data showing that infectivity in the bloodstream is found almost exclusively in the leukocyte fraction [10,11,12,13], while viral DNA in plasma or serum appears to represent mainly free highly fragmented genomes rather than infectious virion particles [14,15].

In fibroblast culture, freshly isolated HCMV almost always spreads in a strictly cell-associated manner, i.e., the virus forms foci of infected cells but no infectivity is detectable in cell-free supernatant [16,17]. Notably, this transmission mode is completely insensitive to antibodies that can efficiently neutralize cell-free virus [3,6]. With continued passage of isolates in fibroblast culture, the strict cell-association is regularly lost, and the appearance of infectivity in the supernatant is associated with genetic alterations in RL13, the UL128 locus and the UL/b’ region of the viral genome [18].

All HCMV strains commonly used in research carry at least some of these mutations and can spread in a cell-free fashion, which is reflected in the formation of less restricted comet-shaped foci. Yet, these strains have apparently also retained the ability to spread in a cell-associated fashion, as inferred from spreading in fibroblasts monolayers in the presence of neutralizing antibodies [5]. This antibody-resistant spread is characterized by the formation of smaller well confined foci and resembles the appearance of foci when supernatant-associated dissemination in the culture is impeded by overlay with methylcellulose or agarose. Whether cell-associated spread, operationally defined by resistance against antibodies or modification by overlay media, is mechanistically identical to cell-associated spread of recent isolates, defined by lack of supernatant-associated infectivity, is unclear.

While few data are available concerning the details of cell-to-cell spread, molecular events for the cell-free transmission of HCMV are well characterized. Productively infected cells release enveloped virions into the environment, which then bind to other cells in the surroundings and can enter these cells by fusion of the viral envelope with cellular membranes, and this process is mediated by a conserved set of herpesviral envelope glycoproteins [19,20,21,22,23]. Homotrimers of gB accomplish the fusion of the viral envelope with cellular membranes, resulting in release of the viral capsid into the cytoplasm of the infected cell. gH/gL complexes are assumed to trigger the fusogenic activity of gB, either as a direct consequence of binding to a cellular receptor or mediated by an additional viral envelope protein that activates gH/gL upon its own interaction with a cellular receptor. The data available to date suggest that both principles apply in HCMV, depending on the cell type. HCMV expresses at least two different variants of gH/gL complexes in its envelope, a trimeric complex of gH/gL with gO as an accessory protein [24,25] and a pentameric gH/gL complex with the three proteins of the UL128 locus as accessory proteins (pUL128, pUL130, and pUL131A) [26,27,28]. The trimer is apparently required for efficient infection of all cell types by cell-free virions [29], whereas the pentamer is additionally required only in certain target cell types, such as endothelial cells, epithelial cells, and leukocytes [28,30]. Fibroblasts appear to be infected via a direct interaction of the trimeric gH/gL/gO complex with its cellular receptor PDGFRα that triggers fusion via gB, whereas endothelial and epithelial cells lack this receptor [31,32,33]. The cellular surface molecule NRP2 was recently suggested as an entry receptor for HCMV on these cell types that can bind to the pentameric gH/gL/pUL128/130/131A complex [34]. For subsequent activation of fusion, however, the trimeric complex is nevertheless required [29], suggesting that only this complex can trigger the fusogenic action of gB.

While the contribution of trimer and pentamer to the cell-free infection mode is well established, their role in cell-associated infection is less clear. Whereas cell-free infection depends greatly on gO irrespective of the cell type [29,35], cell-associated spread can be mediated by either the gH/gL/gO trimer or the gH/gL/pUL128-131A pentamer [36,37,38]. Knockout of both trimer and pentamer, e.g., by combined disruptive mutations in UL74 and one of the UL128-131A genes, completely abrogated the spread of such mutants in fibroblast cultures, which indicates that gH/gL complexes are essential also for cell associated spread, but this has not yet been formally demonstrated. A variant of strain Merlin that has been cloned into a bacterial artificial chromosome (BAC) vector with repaired RL13 and UL128L under the control of a tetracycline operator resembles clinical isolates by growing focally without detectable infectivity in the supernatant [39]. Knockout of gO affected neither focus size nor resistance against antibodies in this virus, while UL128stop mutations increased focus size, and this surplus of focus size was then sensitive to antibodies and associated with some release of cell-free infectivity [6,37,39,40], suggesting that the pentamer restricts viral growth to the cell-associated mode. Quantitative aspect may also play a role as focal spread of the pentamer^high^ strain Merlin in Adult retinal pigment epithelial cells (ARPE-19) cells was resistant against antibodies, whereas focal spread of the pentamer^low^ strain TB40/E and a Merlin mutant with reduced pUL128 expression was sensitive [6], indicating that release of cell-free virus is a matter of pUL128 levels.

Yet, strain Merlin is also distinct from other established HCMV strains by lower trimer levels, which may explain why knockout of gO had no effect in this virus but greatly reduced focal growth in the genetic background of the trimer^high^/pentamer^low^ strain TB40/E [35,36]. Remarkably, antibodies were able to further reduce these small foci, again fitting the idea of virus release under pentamer^low^ conditions [36,41]. A caveat arises from the fact that a splice acceptor site of UL73 is located adjacent to the ATG of UL74; therefore, part of the phenotype of that mutant may be due to effects on the expression of glycoprotein gN [35,42]. Little is known on the role of the gM/gN complex in virus transmission, except that antibodies to gM/gN can neutralize cell-free HCMV [43]. The fact that both components of this complex, as well as gH, gL, and gB, were essential for virus growth in the context of the fibroblast-adapted strains AD169 and Towne [44,45,46] indicates a contribution also to cell-associated growth. However, as those strains lack the pentamer the contribution of each glycoprotein to cell-associated spread should be reexamined in a pentamer^high^ virus that spreads in a strictly cell-associated manner.

Unfortunately, targeted knockout of individual genes is not possible in recent isolates which are closest to the vivo situation, but the genetically repaired BAC-cloned Merlin provides a model of a cell-associated virus that can be mutated, with the additional benefit of reporter genes including Gaussia luciferase [3,39]. With respect to recent isolates, methods based on inhibitory gene-specific RNAs could at least allow the analysis of genes for which partial reduction of expression may provide sufficient information. Therefore, we used the Merlin-BAC to evaluate how knockout of gH, gL, gB, gM, and gN affects cell-associated spread, and attempted to assess the role of trimer and pentamer by siRNA-mediated knockdown of UL74 and UL128 in recent isolates. Specifically, we tested the hypothesis that partial reduction of pentamer expression would lead to release of cell-free infectivity from strictly cell-associated HCMV isolates as suggested by experiments in the background of strain Merlin [6], and that residual expression of the pentamer would allow infection of epithelial cells, thus resembling the situation with trimer^high^/pentamer^low^ strains, like TB40/E. If so, this would not only provide insight into the role of gH/gL-complexes in cell-associated spread but could also facilitate the transfer of recent isolates from fibroblast cultures to other cell types without the need for coculture.

## 2. Materials and Methods

### 2.1. Cells

For propagation, human foreskin fibroblasts (HFF) were kept in “growth medium” containing minimal essential medium with 5% fetal bovine serum (PAN Biotech, Aidenbach, Germany) GlutaMAX (Life Technologies, Carlsbad, CA, USA), 100 µg/mL gentamicin, and basic fibroblast growth factor (bFGF; Life Technologies, 0.5 ng/mL). Human fetal foreskin fibroblasts (HFFF-tet) cells were immortalized with hTERT and expressed the Tet-repressor [39]. For propagation, HFFF-tet cells were cultured in “growth medium”. During experiments, both HFFs and HFFF-tet cells were kept in “growth medium” without bFGF (MEM5). Human umbilical vein endothelial cells (HUVECs) were cultured in RPMI1640 medium (Life Technologies) supplemented with 10% HCMV-seronegative human serum, 50 µg/mL endothelial cell growth supplement (ECGS, BD Biosciences, Franklin Lakes, NJ, USA), 5 units/mL heparin (Sigma-Aldrich, St. Louis, MO, USA), and 100 µg/mL gentamicin. Adult retinal pigment epithelial cells (ARPE-19) were cultured in DMEM/F-12 with GlutaMAX (Gibco, Thermo Fisher Scientific, Waltham, MA, USA) supplemented with 5% FBS and 100 µg/mL gentamicin. Cell-culture microplates were coated with 0.1% gelatin (Sigma-Aldrich) prior to seeding of cells.

### 2.2. Viruses

Merlin pAL1502 is a bacterial artificial chromosome (BAC)-cloned HCMV strain that is a derivative of the repaired Merlin-BAC pAL1128 [6,39]. It has tet-operator sequences in front of the RL13 and UL128 genes. Merlin pAL1502-GLuc is a derivative of Merlin pAL1502 that expresses Gaussia luciferase under control of the major immediate early (IE) promotor/enhancer [3]. TB40-BAC4 is a BAC-clone based on HCMV strain TB40/E [47], TB40-BAC4-UL74stop is a derivative thereof which lacks the expression of pUL74 (gO) [35] and TB40-BAC4ΔUL132-128 is a derivative that lacks the complete UL128 gene region [48].

Recent clinical HCMV isolates were provided by the diagnostic laboratory of the Institute of Virology in Ulm. They originated from routine testing of throat washings from patients of the Ulm University Medical Center. Sample material was applied to HFFs, and HCMV-positive cultures were then incubated for several weeks until they showed the desired cytopathic effect. Infected cells were then aliquoted and frozen at −80 °C, and the cell association of the HCMV isolates was tested by transferring culture supernatants onto adherent HFFs and immunofluorescence staining for viral immediate-early (IE) antigens one day after inoculation. Isolates were only used further if they were negative in this assay. 

For preparation of cell-free virus stocks, supernatants from productively infected cultures were centrifuged for 10 min at 2790× *g* to remove cells and debris and stored at −80 °C in aliquots until used in experiments. For preparation of purified virions, cell-free supernatants were fractionated by gradient centrifugation [49]. First, HCMV particles were pelleted from cell-free supernatants by ultracentrifugation at 70,000× *g* for 70 min, and the pellets were resuspended in 2 mL sodium phosphate buffer (8 mM NaH_2_PO_4_, 32 mM Na_2_HPO_4_, pH 7.4). This suspension was then layered on a linear glycerol-tartrate gradient (15% sodium tartrate/30% glycerol to 35% sodium tartrate in phosphate buffer) and centrifuged at 80,000× *g* for 45 min, resulting in separation of HCMV particles into non-infectious enveloped particles (NIEPs), virions, and dense bodies. The virion fraction was collected by syringe and needle, resuspended in phosphate buffer, and recentrifuged at 80,000× *g* for 70 min. The supernatant was discarded, and virion pellets were stored at 80 °C until used for Western blot analyses.

### 2.3. Generation of Mutant Viruses

Mutant BACs were generated by applying a markerless mutagenesis protocol [50]. In brief, using plasmid pEP-Kan-S as a template recombination fragments were generated by PCR that consisted of the 18-bp I-Sce I restriction site and a kanamycin resistance cassette flanked by repeated sequences containing homology to the desired site of insertion in the HCMV genome. Since the primers containing the homology regions (Table 1 and Table 2) showed a high binding potential to each other, the recombination fragment was synthesized in two separate PCR reactions. Using the forward primer and the kanamycin universal reverse primer, the kanamycin cassette region and the I-SceI restriction site of the plasmid were amplified. The resulting fragment was used as a template for a second amplification with the short-forward and reverse primers to obtain the final recombination fragment, which was then inserted by electroporation into recombination-activated GS1783 harboring the Merlin pAL1502-GLuc BAC or Merlin pAL1502. Following kanamycin selection, all non-HCMV sequences were removed by intrabacterial I-Sce I digestion and a subsequent red recombination step. BAC-DNA was isolated using the NucleoBond Xtra Midi kit (Macherey-Nagel, Düren, Germany), each mutant was analyzed by RFLA and Sanger sequencing, and to reconstitute the virus, the purified BAC-DNA was transfected into HFFFtet cells using a calcium phosphate-based method (MBS Transfection Kit, Agilent, Waldbronn, Germany) or lipofection (K2 Transfection System, Biontex Laboratories, München, Germany).

All primers were designed to match the sequence of both TB40-BAC4 and Merlin, and with each primer set two mutant clones were generated in TB40-BAC4 in order to test whether the stop codons abolished virus growth as expected. In UL73, these control experiments showed that two stop codons were not sufficient to fully prevent viral growth. Hence another doublet of stop codons was inserted including a stop codon at the position of the third methionine in the amino acid sequence of UL73. With this additional mutation, virus growth was abolished consistent with the previous findings of UL73 being essential for replication of cell culture adapted strains. The same primer sets were then applied to the genetic background of strain Merlin using Merlin-BAC pAL1502-GLuc, which allowed the evaluation of viral growth during the reconstitution procedure by measuring luciferase activity in the supernatant of transfected cultures.

### 2.4. Determination of Viral Growth

The use of Gaussia luciferase for quantification of infection has been described previously [3]. Briefly, the Gaussia luciferase-containing cell culture supernatants were either stored at −20 °C or mixed immediately with the luciferase substrate coelenterazine (PjK, Kleinblittersdorf, Germany). Coelenterazine was diluted to 0.2 µg/mL in phosphate buffered saline with 5 mM NaCl. The substrate was added to the cell culture supernatants automatically in a plate reader (Chameleon, Hidex, Mainz, Germany) and the luminescence signals were measured as relative light units (RLU). The RLU-values of the luciferase activity were plotted on a logarithmic scale against the time after transfection to visualize the viral growth in the cell culture. The slope of the curve during the phase of exponential growth, appearing linear in the logarithmic scale, was used to calculate the increase of virus (growth rate = 10^slope^ − 1).

### 2.5. Knockdown of Gene Expression with siRNA

HFFs were transfected with 200 nmol/l siRNA using Lipofectamine RNAiMAX transfection reagent (Life Technologies). For knockdown of the cellular genes targeting the cellular genes PDGFRα and NRP2 premixed pools of four siRNAs were used (M-003162-04 and M-017721-01; Thermo Fisher Scientific). For knockdown of the viral genes UL74 and UL128, two individual siRNAs per gene were designed and purchased from Sigma-Aldrich: UL74 was targeted by 5′-CGAACAAGGCUGCGGUAAU(dT)(dT)-3′ and 5′-GGUCCCAUUCGAAACGAUA(dT)(dT)-3′, UL128 was targeted by 5′-GCGGCAAAGUGAACGACAA(dT)(dT)-3′ and 5′-CUGCUACAGUCCCGAGAAA(dT)(dT)-3′. To control for unspecific effects of siRNA transfection, a pool of non-targeting (NT) siRNAs was included (D-001206-14; Thermo Fisher Scientific).

### 2.6. Detection of Viral Immediate Early Proteins by Indirect Immunofluorescence

Infected cells were fixed with 80% acetone for 5 min at room temperature, washed with PBS, incubated at 37 °C for at least 90 min with antibody E13 (Argene/Biomerieux, Marcy-l’Étoile, France) directed against viral immediate early (IE) antigen (UL122/123), washed with PBS, incubated for 60 min with Cy3-goat-anti-mouse Ig F(ab’)2 fragments (Jackson ImmunoResearch, West Grove, PA, USA), washed with PBS, and counterstained with 4′,6-Diamidin-2-phenylindol (DAPI, Sigma-Aldrich). This procedure resulted in nuclear red fluorescence of infected cells and blue nuclear fluorescence of all cells. Images were taken fluorescence microscopy with an Axio Observer D1 microscope (Zeiss, Oberkochen, Germany), and, if desired, quantification was done using Zen software (version 2.3, Zeiss).

### 2.7. Immunoblotting

Samples were lysed in Laemmli lysis buffer [51], the lysates were boiled for 5 min at 95 °C, precipitates were removed by centrifugation, and the cleared lysates were stored in aliquots at −80 °C. For immunoblotting under reducing conditions, lysates were thawed, boiled with 5% β-mercaptoethanol for 5 min at 95 °C, and clarified from precipitates by centrifugation, whereas for nonreducing conditions, β-mercaptoethanol was omitted. Lysates were then loaded onto 10% or 12.5% polyacrylamide gels and electrophoresis was performed in tris glycine SDS buffer. The proteins were transferred onto PVDF membranes in tris-glycine buffer with 15% methanol. Membranes were blocked with PBS plus 0.1% Tween and 5% milk powder before staining with rabbit antibodies against actin (Sigma-Aldrich) or mouse monoclonal antibodies against the viral proteins gO [37], gB (Abcam, Cambridge, UK), pUL128 [52] or the cellular protein PDGFRα (Cell Signaling Technology, Danvers, MA, USA). Horseradish peroxidase (HRP)-conjugated rabbit anti-mouse-Ig (Agilent DAKO, Santa Clara, CA, USA) or goat anti-rabbit-Ig (Santa Cruz Biotechnology, Dallas, TX, USA) was used as secondary antibody. Visualization and quantification of the signals was performed with Super Signal West Dura Extended Duration substrate (Thermo Fisher Scientific) using FusionCapt Advance Solo (v.7 Vilber Lourmat, Eberhardzell, Germany).

### 2.8. Statistical Analyses

**Figure 1:**
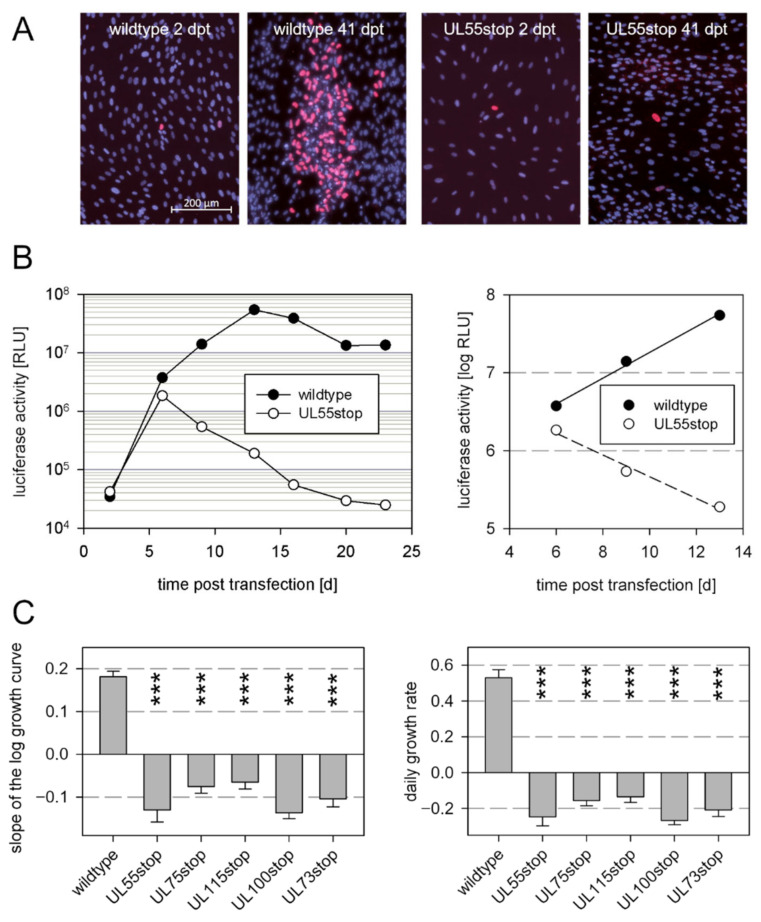
Effect of stop mutations in viral genes encoding for envelope glycoproteins on growth of the cell-associated model virus Merlin pAL1502-GLuc. (**A**) Detection of viral immediate early antigen 2 d post-transfection (dpt) as a proof of successful transfection and 41 dpt as a readout of viral growth. Data for the UL55stop mutant are shown as an example. (**B**) Measurement of Gaussia luciferase activity (relative light units; RLU) as a reporter for viral gene expression. The period during which growth dynamics were exponential and appear linear on a logarithmic scale were used for linear regression analyses to determine the slope as a readout for viral growth. Data for the UL55stop mutant are shown as an example. (**C**) Growth dynamics of Merlin pAL1502-GLuc (wildtype virus) and glycoprotein-stop-mutants thereof. The daily growth rates (right panel) were calculated from the slopes (left panel) as 10^slope^ − 1. Bars indicate mean values of eight replicates (two measurements of two independent transfections of two clones per gene). Error bars represent the standard error of the mean (SEM). Asterisks indicate significant differences as compared with wildtype (***, *p* < 0.001).

**Figure 2:**
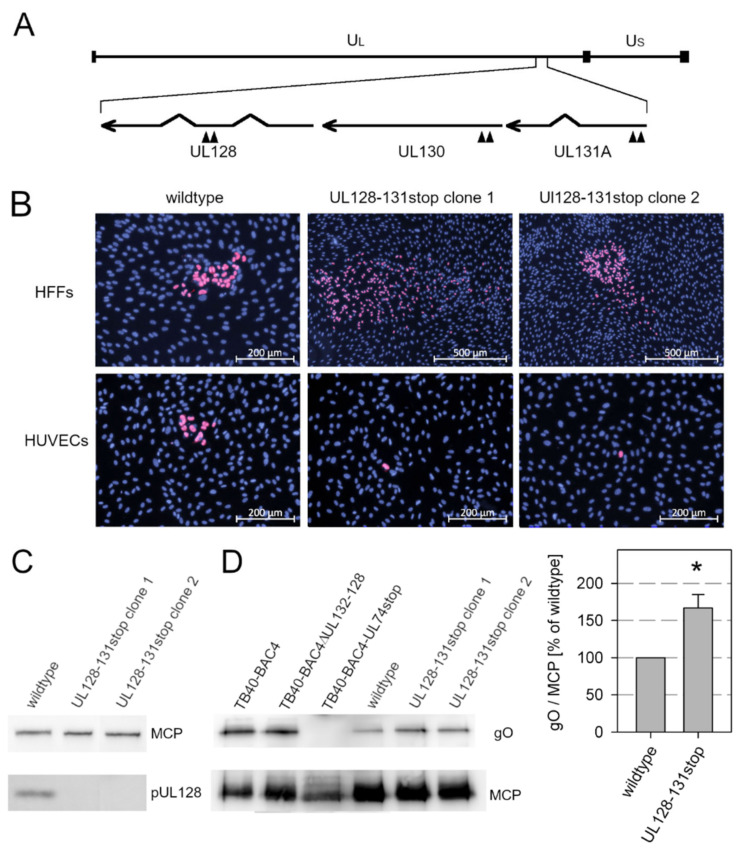
Effect of stop mutations in viral genes encoding for the accessory proteins of the pentamer complex. (**A**) Schematic representation of the viral genome with the unique long and the unique short section indicated by UL and US, respectively. The UL128 gene locus is shown enlarged below the genome map, and the positions of the stop codons that were introduced into the open reading frames of UL128, UL130, and UL131A are indicated by arrow heads. (**B**) Virus reconstituted in HFFFtet cells from wildtype Merlin pAL1502 or mutant genomes was used to infect fibroblasts (HFFs) which were then cocultured with an excess of uninfected HFFs or endothelial cells (HUVECs) for 5 d. After the incubation time, cultures were fixed and immunostained for viral immediate early (IE) antigen. (**C**) Lysates of infected HFFs were analyzed by separation on a 10% polyacrylamide gel under reducing conditions and subsequent immunoblotting with antibodies against pUL128 to test whether the knockout of UL128 was successful. The major capsid protein (MCP) was detected as a loading control. (**D**) Virus particles were prepared from infected HFFs (TB40-BAC4 and derivatives) or HFFFtet cells (Merlin pAL1502 wildtype and UL128-131stop mutants) by gradient purification and lysed. Lysates were separated on a 10% polyacrylamide gel under nonreducing conditions and analyzed by immunoblotting with antibodies against glycoprotein O (gO) or MCP (loading control). For Merlin wildtype and mutants, the ratio of gO/MCP signals were determined to evaluate the effect of the knockout of the UL128 locus genes on the incorporation of gO into virion particles. Bars indicate mean values of four replicates with wildtype (two blots of two independent preparations) and eight replicates of UL128-131stop (two blots of two independent preparations of each of the two clones). Error bars represent the standard error of the mean (SEM). The asterisk indicates a significant difference as compared with wildtype (*, *p* < 0.05).

Datasets with more than two groups of data were analyzed by one-way-ANOVA using the build-in data analyses function of Sigmaplot to test whether there are significant differences between the various conditions. If ANOVA indicated significant differences between groups within the data set, appropriate post-hoc analyses were performed to identify groups that differ from the untreated control (Holm-Sidak for data sets in Figures 1 and 4B; paired t-tests for data sets in Figures 5 and 6). Datasets in Figures 2 and 4A were analyzed using unpaired t-tests. Differences between conditions were considered marginally significant when *p*-values were <0.05, significant when *p* values were <0.01 and highly significant when *p* values were <0.001.

## 3. Results

### 3.1. Envelope Glycoproteins That Are Essential for Cell-Free Virus Spread Are Also Essential for Cell-Associated Growth

As the essential role of the glycoproteins gB, gH, gL, gM, and gN has only been proven in the context of viruses that lack the pentameric gH/gL complex and grow via the cell-free mode [44,45,46], we aimed to revisit the contribution of these glycoproteins in the genetic background of a virus that grows strictly cell-associated, like recent clinical HCMV isolates. To knock out expression of the individual glycoproteins, we introduced stop codons into the respective open reading frames of a genetically repaired variant of strain Merlin that is available as a BAC-cloned genome and hence allows for targeted genetic manipulation [39]. This virus grows strictly cell-associated due to expression of RL13 and the UL128 locus and, therefore, allows the evaluation of whether the herpesviral fusion machinery is also essential under these conditions.

All genome modification were introduced into the bacterial artificial chromosome (BAC) Merlin pAL1502-GLuc [3] in E.coli using the seamless mutagenesis approach by Tischer et al. [50], and for reconstitution of the recombinant viruses BAC DNA was isolated from the mutated bacteria and transfected into fibroblasts. To impede accidental repair of introduced stop mutations during the reconstitution process, we always introduced a set of two stop codons (Table 1). To ensure that the reconstitution process worked, wildtype BAC Merlin pAL1502-GLuc was always included as a positive control. The transfection efficiency was controlled by immunofluorescence detection of viral immediate early antigens (Figure 1A), and reconstitution of virus from the transfected genomes was monitored by measurement of luciferase activity in the supernatant of the transfected cultures Figure 1B). The log fold change of luciferase signals during the exponential growth phase was calculated, and the growth rate per day was calculated from this slope value. Two measurements were taken for each of two independent transfections with each of the two clones, resulting in eight values for each mutation, which were then used for statistical analyses in comparison to wildtype virus in an ANOVA with appropriate post-hoc tests.

As expected, viable virus could be reconstituted by transfection of wildtype genomes, reflected by an exponential increase of the luciferase signals (Figure 1B). In contrast, virus growth was not detectable with any of the mutant genomes (Figure 1C). Transfection efficiencies with the mutants were comparable to wildtype controls as reflected by the number of cells expressing viral IE antigen and an initial luciferase expression. However, the level of luciferase signals declined after the initial signal peak and subsequently disappeared, indicating that viable virus could not be reconstituted. This set of data confirmed the essential role of gB, gH, gL, gM, and gN in the background of a strictly cell-associated HCMV strain.

### 3.2. Knockout of the Pentamer Has Only Limited Effect on Expression of the Trimer in the Background of Strain Merlin

While gH and gL were both required for Merlin growth, each of the two alternative gH/gL complexes appeared to be dispensable for growth in fibroblast cultures in previous studies [37,39]. In the genetic background of strain Merlin, deletion of the trimer by a dual-stop mutation in UL74 only slightly affected focal growth, and deletion of the pentamer by a dual-stop mutation in UL128 even enhanced viral spread. In contrast, combined mutations of UL74 and one of the UL128-131A proteins were lethal to the virus, consistent with the assumption that gH/gL complexes are essential and gH/gL dimers are insufficient for viral replication in the absence of accessory proteins [37]. However, the unrestricted growth of the UL128 stop mutant was somewhat surprising, as Merlin is known to express very low levels of trimers, even under conditions where expression from the UL128 locus is greatly reduced [53]. On the other hand, Merlin expresses particularly high levels of pentamer [40], and we wondered whether only the complete absence of UL128-131A proteins could lead to trimer expression comparable to trimer^high^ strains, such as TB40/E. To formally exclude any competition for gH/gL, we constructed a new mutant of Merlin pAL1502 with two stop codons in each of the UL128 locus genes (Figure 2A).

To exclude the possibility that the phenotype of the mutant virus may be due to an accidental unintended second site mutation, two independent clones were generated and analyzed. Virus was reconstituted from the mutant genomes by transfection into HFFFtet cells, and sequence analysis of the reconstituted viruses confirmed that the six stop mutations had been introduced as intended and no unwanted mutations had occurred in the UL128 locus. The reconstituted viruses were transferred to normal HFFs, which were then cocultured with an excess of uninfected HFFs or endothelial cells (HUVECs) for 5 d. Both clones showed the expected phenotype (Figure 2B), i.e., mutants formed comet-shaped foci, indicating release of cell-free infectivity, whereas wildtype Merlin pAL1502 was strictly cell-associated, and both mutants did not form foci in endothelial cell cultures, whereas the wildtype virus spread focally. Concordant with this phenotype, pUL128 signals were not detected in immunoblotting analyses of lysates from infected HFFs, whereas lysates of wildtype virus showed the expected band (Figure 2C). Two independent virion preparations of each virus were then generated by glycerol tartrate gradient centrifugation from supernatants of infected HFFFtet cultures and compared in nonreducing immunoblotting analyses for incorporation of the gH/gL/gO trimer. As hypothesized, complete knockout of the UL128-UL131A proteins further increased the expression of gO above the level in wildtype virus produced under conditions of UL128 repression (Figure 2D). However, the increase was only moderate to 167% of wildtype level (standard error of the mean = 22%, *p* = 0.029) and did not reach the levels detected with strain TB40/E. At this point, we wondered whether strain Merlin is unique or whether it shares the expression pattern with recent clinical isolates that also grow strictly cell-associated.

### 3.3. Recent Isolates of HCMV Express Higher Levels of gO Than the Cell-Associated Model Virus Merlin pAL1502

To address the question of which of the laboratory strains TB40/E and Merlin more better represents clinical isolates regarding expression of the alternative gH/gL-complexes, we prepared lysates of recent clinical isolates grown in HFF cultures and analyzed them by quantitative immunoblotting for the amount of gO and pUL128 as indicator proteins for trimer and pentamer. The isolates were selected for a strictly cell-associated phenotype, i.e., lack of infectivity in the supernatant, and an infection rate in the isolate culture of at least 25% to allow detection of viral proteins in immunoblotting. A total of 8 isolates were included, and three independent lysates of each isolate were analyzed by immunoblotting under reducing conditions using monoclonal antibodies against gO, pUL128 and gB. The latter was used as a reference protein in densitometric analyses of the resulting bands, and gO/gB and UL128/gB ratios were used as readouts for the relative amount of gO and pUL128. For comparison, three lysates of Merlin pAL1502 and TB40/E were included, representing a cell-associated trimer^low^/pentamer^high^ and a cell-free trimer^high^/pentamer^low^ lab strain, respectively.

**Figure 3:**
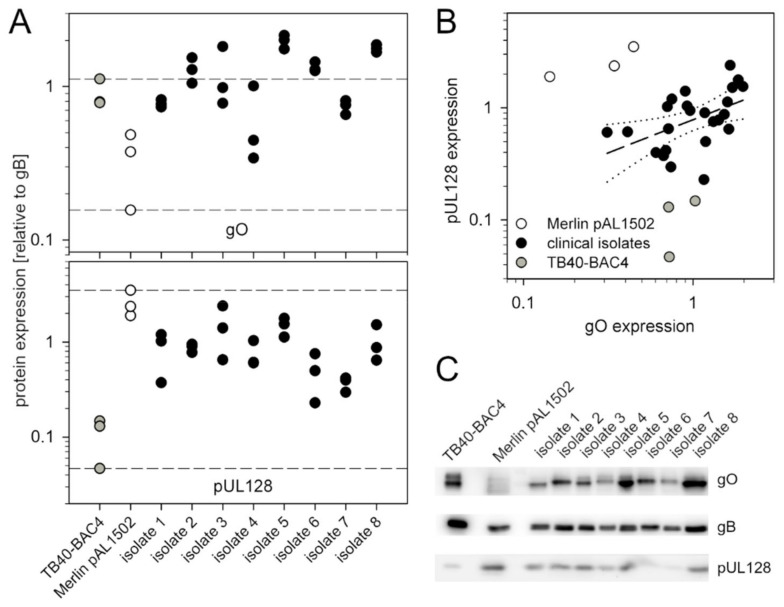
Comparison of various recent clinical human cytomegalovirus (HCMV) isolates and virus strains TB40/E and Merlin regarding the relative abundance of glycoprotein O and pUL128. (**A**) For each isolate or virus strain, three independent lysates of infected cell cultures were analyzed by separation on 10% polyacrylamide gels and subsequent immunoblotting for glycoprotein O (gO), pUL128 and glycoprotein B (gB) using monoclonal antibodies against the respective protein. gB was used as a reference protein, and ratios of gO/gB signals and pUL128/gB signals were used as a readout for the relative abundance of gO and pUL128. Each dot represents one lysate. Dashed lines indicate the upper and lower limits of the values for laboratory strains TB40/E and Merlin. (**B**) Values of the relative abundance of pUL128 and gO were plotted against each other, and for clinical isolates (black dots) a linear regression analysis was performed (dashed line) Dotted lines represent the 95% confidence interval. (**C**) An example of one representative immunoblotting analysis underlying the analyses of (**A**,**B**) is shown.

The reproducibility of gO/gB and pUL128/gB ratios in the three lysates was sufficient to detect differences between the various viruses (Figure 3A). Regarding gO, the expression level was remarkably high, with four of the isolates even exceeding the level of the trimer^high^ strain TB40/E and all except one expressing more gO than strain Merlin. Regarding pUL128, all isolates expressed higher levels than the pentamer^low^ strain TB40/E, and none of the isolates exceeded strain Merlin. When the pUL128/gB were plotted against the gO/gB values (Figure 3B), it became clear that the laboratory strains represented extremes that were clearly distinct form the isolate data by either a pentamer^low^ or a trimer^low^ phenotype. Within this population, the correlation between pUL128/gB and gO/gB values was not very strong, but clearly positive, i.e., isolates that expressed higher levels of pUL128 also expressed higher levels of gO.

In conclusion, neither of the lab strains appeared to represent clinical isolates regarding expression of the accessory proteins of pentamer and trimer, with most of the clinical isolates showing a pentamer^high^/trimer^high^ phenotype.

### 3.4. Spread of Recent Isolates in Fibroblast Culture Is Promoted by gO and Restricted by pUL128

While an essential role of gO for entry of cell-free HCMV virions into various target cell types is well established, the contribution of gO to cell-associated spread is less clear. On one hand, deletion of gO in the background of the cell-associated BAC-cloned strain Merlin had little effect on focal growth if the alternative pentameric gH/gL complex was intact [37]. On the other hand, analysis of gO in the Merlin background may underestimate the role of gO as this protein is only inefficiently incorporated into the gH/gL/gO trimer in this strain [53,54]. In the light of our novel finding that recent isolates express significantly more gO than Merlin it appeared appropriate to revisit the role of gO for focal spread in the context of these isolates.

**Figure 4:**
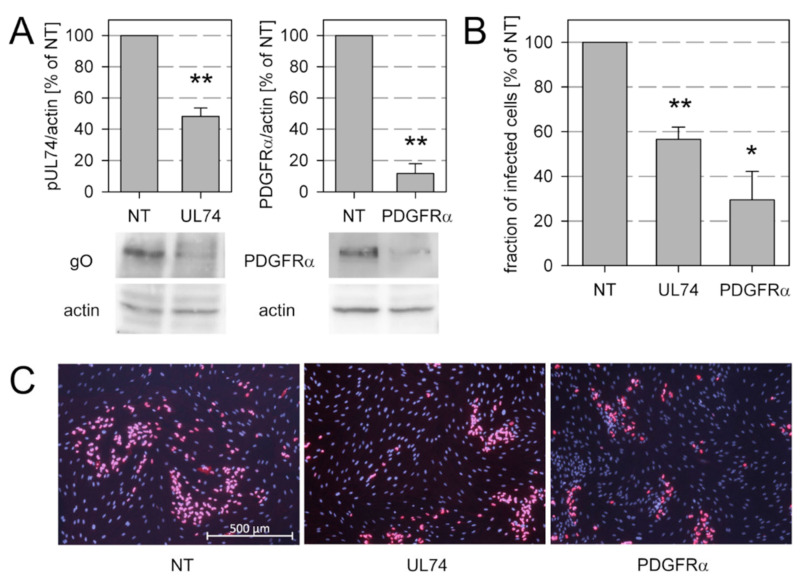
Knockdown of viral glycoprotein O (gO) and its cellular receptor PDGFRα both reduce the growth of cell-associated clinical HCMV isolates. Human fibroblasts infected with recent HCMV isolates were transfected with siRNAs targeting transcripts of the viral gene UL74 (encoding gO) or the cellular gene encoding the receptor PDGFRα. Nontargeting siRNAs (NT) were included as a control. (**A**) Four days after transfection, cultures were lysed and analyzed by immunoblotting for expression of gO or PDGFRα, including detection of actin as a loading control. Chemiluminescence signals were analyzed by densitometry, and UL128/actin ratios were calculated and normalized to the NT controls. (**B)**,**C**) Replicates of the transfected cultures were fixed and immunostained for viral immediate early (IE) antigen as an indicator of viral growth. Both knockdown of gO and knockdown of PDGFRα inhibited viral growth to a degree that corresponded to the expression levels of the proteins after siRNA treatment. Bars indicate mean values of three biological replicates (different isolates). Error bars represent the standard error of the mean (SEM). Asterisks indicate significant differences as compared with NT (**, *p* < 0.01; *, *p* < 0.05).

To address this issue, we knocked down gO expression in the context of recent HCMV isolates by transfection of two pooled UL74-specific siRNAs and evaluated the effect of this knockdown on the extent of focal growth, using non-targeting siRNAs as a negative control. In parallel, we also knocked down the cellular receptor of gO, PDGFRα, that was reported to promote cell-associated spread of the laboratory strains VR1814 and TB40-BAC4 [33] but has not been analyzed in the context of recent clinical isolates. The efficacy of siRNA-mediated knockdown was tested by quantitative western blot analyses, showing a 50% reduction of gO expression and an almost complete knockdown of PDGFRα expression as compared to non-targeting siRNA (Figure 4A). Both, gO and PDGFRα had an effect on focal growth (Figure 4B,C). The partial knockdown of gO reduced growth of the various isolates by 43.5%, while the more complete knockdown of PDGFRα reduced viral growth by 70.5%. These results supported the hypothesis that the cell-associated virus spread of recent clinical isolates in fibroblast cultures is promoted by an interaction of the trimeric complex gH/gL/gO with its cellular receptor PDGFRα.

**Figure 5:**
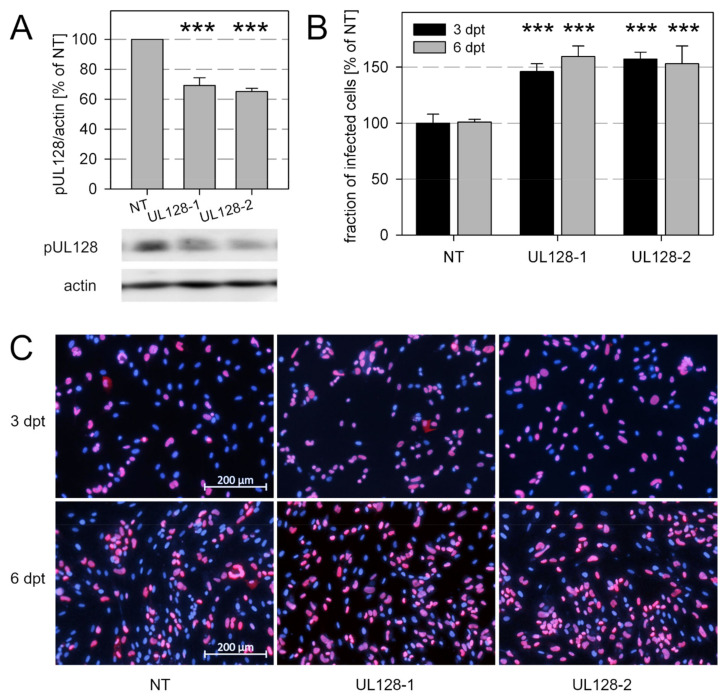
Knockdown of UL128 promotes growth of clinical HCMV isolates. Human fibroblasts, infected with recent HCMV isolates, were transfected with two individual siRNAs targeting transcripts of the viral gene UL128. Nontargeting siRNAs (NT) were included as a control. (**A**) Six days after transfection (dpt), cultures were lysed and analyzed by immunoblotting for expression levels of UL128, including detection of actin as a loading control. Chemiluminescence signals were analyzed by densitometry, and UL128/actin ratios were calculated and normalized to the NT controls. Bars indicate mean values of six replicates (three independent transfections with two isolates). Error bars represent the standard error of the mean (SEM). The asterisks indicate significant differences as compared with NT controls (***, *p* < 0.001). (**B**) Replicates of the transfected cultures were fixed 3 or 6 dpt and immunostained for viral immediate early (IE) antigen as a readout for viral growth. The fraction of infected cells was determined and normalized to the NT controls. Bars indicate mean values of six replicates (3 dpt: six technical replicates, 6 dpt: two independent transfections with three isolates). Error bars represent the standard error of the mean (SEM). The asterisks indicate significant differences as compared with NT controls (***, *p* < 0.001). (**C**) Representative examples of the immunostainings analyzed in Panel B.

To assess the role of the pentamer in the same setting we next knocked down expression of its accessory protein pUL128 by two UL128-specific siRNAs. With both siRNAs, the expression of pUL128 was reduced by about one third as compared to the nontargeting control-siRNA (Figure 5A). This knockdown was associated with an increased spread of the various isolates in the culture (Figure 5B,C). 

### 3.5. Knockdown of pUL128 Switches HCMV Isolates Transiently to the Cell-Free Transmission Mode

**Figure 6:**
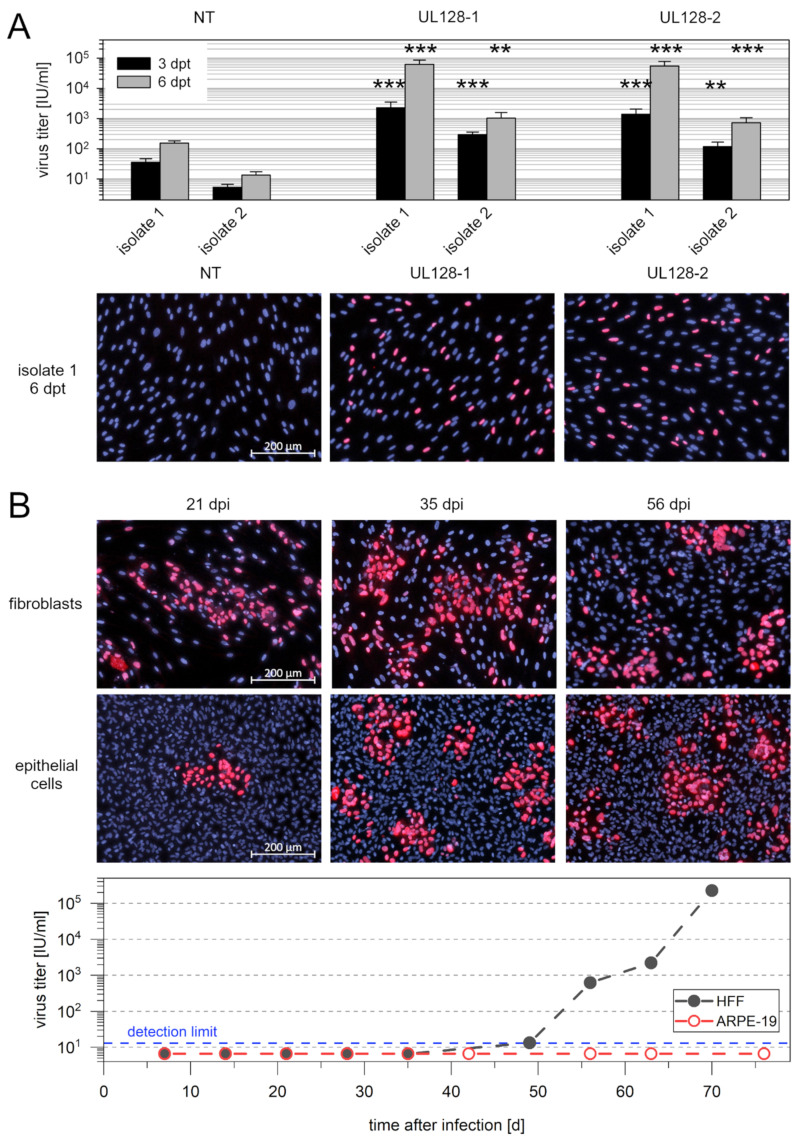
Effect of siRNA-mediated knockdown of pUL128 on release of cell free infectivity from recent clinical HCMV isolates. Human fibroblasts (HFFs), infected with recent HCMV isolates, were transfected with two individual siRNAs targeting transcripts of the viral gene UL128. Nontargeting siRNAs (NT) were included as a control. (**A**) Three days or six days after transfection (dpt), supernatants of the transfected cultures were harvested, clarified from cells and debris, and tested for infectivity. For this, the cell-free supernatants were incubated with uninfected HFFs overnight, fixed and immunostained for viral immediate early (IE) antigen. The fraction of infected cells was determined, and the corresponding virus titer in the respective supernatant was calculated. Bars indicate mean values of 4–6 replicates. Error bars represent the standard error of the mean (SEM). The asterisks indicate significant differences as compared with NT controls (***, *p* < 0.001; **, *p* < 0.01). Representative examples of the immunostainings are shown for 6 dpt. (**B**) Supernatants of isolate 1 were transferred to infect fibroblasts (HFFs) and epithelial cells (ARPE-19) for long-term propagation. Every week, the cell cultures were passaged, and cell-free supernatants were harvested and analyzed for infectivity. A small fraction of the passaged cells was cultured separately, fixed after one week and immunostained for viral IE antigen to evaluate the potential for focal growth. Examples of focus formation are shown in both cell types for passages at 3, 5 and 8 weeks after the initial transfer. Starting from week 7, cell-free infectivity became detectable in HFFs but not in ARPE-19 cells.

The increase in virus spread that we observed after knockdown of UL128 could be due to either increased efficiency of cell-to-cell spread or additional supernatant-associated spread. The latter appeared likely since the complete knockout of UL128-131A expression is well known to result in release of cell-free infectivity. Furthermore, the cell-free phenotype of pentamer^low^ strains, like TB40/E, VHL/E, and VR1814, indicates that not only complete loss but also reduced expression from the UL128 locus can be associated with a cell-free phenotype [40]. Hence, we hypothesized that the partial knockdown of UL128 expression by treatment with siRNA abrogates the strict cell-association of clinical HCMV isolates and leads to release of infectious virus into the supernatant. Therefore, we tested cell-free supernatants obtained from the UL128-siRNA-treated isolate cultures for infectivity by incubating them overnight with fibroblast cultures and detecting viral immediate early antigens via immunofluorescence. Already at day 3 after transfection, we found a 25–65-fold increase in cell-free infectivity compared with nontargeting siRNA, which was further augmented to a 50–400-fold increase on day 6 (Figure 6A).

As the effect of siRNA-mediated knockdown is transient and isolates are not expected to be genetically altered by siRNA treatment, we assumed that the release of cell-free infectivity was also transient and that the virus would switch back to a cell-associated phenotype after transfer to other cell cultures. Therefore, we transferred supernatants from day 6 after transfection to HFFs and ARPE-19 cells for long-term propagation and tested the supernatants for cell-free infectivity at each subpassage. We were interested in whether infection of epithelial cells (ARPE-19) with supernatant of UL128-siRNA-treated isolates was possible despite knockdown of the pentamer, and we expected that if the transfer was successful, the virus could then grow in this cell type in long-term culture. As predicted, the virus grew strictly cell-associated in HFFs after transfer until the 7th passage, when cell-free infectivity became detectable and continued to increase until the 10th passage (Figure 6B). Infection of ARPE-19 cells was possible with UL128 knockdown virus, albeit with very low efficiency, and the virus grew focally in this cell type without evidence of cell-free infectivity throughout the course of the experiment. To analyze the stability of the UL128 gene locus during this experiment, we amplified each of the three open reading frames from samples of (i) the isolate in HFFs before knockdown of UL128, (ii) HFFs 11 d after infection by transfer of the released cell-free virus, and (iii) ARPE19 cells after long-term propagation of the transferred virus for 10 weeks. Using these amplification products, we determined the DNA sequence of the UL128, UL130, and UL131A open reading frames and found no differences between samples. All open reading frames appeared intact and encoded full-length proteins (Figures S1 and S2).

## 4. Discussion

Our finding that partial knockdown of the viral UL128 gene leads to the release of cell-free infectious virus from otherwise cell-associated recent clinical HCMV isolates not only demonstrates the contribution of this gene locus to the regulation of the transmission mode of HCMV, but also has practical implications as it can greatly facilitate work with recent isolates.

The importance of the UL128 gene locus for the cell-associated phenotype was first suggested when disruption of the respective reading frames was found regularly during adaptation of recent HCMV isolates to growth in fibroblasts and these genetic alterations were associated with an increase in virus titers [18]. Further evidence came from attempts to repair the genetic defects in this gene region in BAC-clones of strain Merlin, which showed that presence of an intact UL128 locus reduced cell-free infectivity by approximately three log levels compared with UL128 mutant virus [39]. This matches well with the 50–400-fold increase of cell-free infectivity that we found 6 d after partial knockdown of UL128 in isolate-infected cultures. With this, our knockdown experiments not only provide formal evidence that the initially observed association between the increase in cell-free viruses and disruption of the UL128 locus is a causal relationship in HCMV isolates, but they also add a quantitative aspect, as they suggest that a certain level of UL128 expression is required to keep the virus restricted to cell-associated growth. Whether this is mediated directly by pUL128 or indirectly by the effect on trimer expression still needs to be resolved. With respect to the biology of HCMV in vivo, one might speculate that HCMV can change its transmission mode upon modulation of expression of the UL128 locus genes. For example, reduced expression of the pentamer in epithelial cells of the breast and the kidney could promote the release of cell-free virus in breast milk or urine for transmission to another host.

The available data on the role of UL74, which encodes gO, were less clear. Knockout of UL74 expression in the context of the trimer^high^ strain TB40/E provided circumstantial evidence that gO contributes to cell-associated spread, defined as serum-resistant focal growth [36], whereas UL74 stop mutations in in the background of the trimer^low^ strain Merlin had no significant effect [37]. Our finding that both knockdown of gO and knockdown of the cellular gO-receptor PDGFRα affect focal growth in the background of recent isolates supports the notion that the molecular interaction between these two proteins not only mediates entry of cell-free HCMV [31,32,33] but also promotes cell-associated spread of this virus in fibroblasts. It seems likely that this interaction was overlooked in the background of strain Merlin simply because the low levels of gO in this strain may contribute little to cell-associated growth in the presence of an intact UL128 locus. Presuming that our gO-expression data with eight recent isolates reflect the in vivo situation, it stands to reason that gO can also contribute to cell-associated spread of HCMV in vivo in certain cell types.

The essential role of gH, gL, and gB in the context of strain Merlin strongly supports the idea that both cell-free entry and cell-to-cell-transmission depend on gB-mediated membrane fusion triggered by gH/gL complexes. While this has already been indirectly suggested by the lethal effect of deletion of these genes in the background of the laboratory strains AD169 and Towne, it has now been formally proven in a strain that spreads only via the cell-associated pathway. However, this does not clearly elucidate the exact mechanism of cell-associated spread. Either enveloped virions could be released in a localized manner and fuse their envelopes directly with neighboring cells or, alternatively, the gH/gL complexes and gB could promote fusion between the plasma membranes of infected and uninfected cells, allowing direct transfer of subviral particles. Recent data obtained with a dual fluorescent of strain Merlin favor the first explanation as capsid and envelope signals were colocalized on neighbors of productively infected cells in growing foci [3]. Regardless of which explanation is correct, the necessity of the fusion machinery for cell-to-cell spread provides a rationale for finding fusion inhibitors that can block both modes of transmission by the same molecular mechanism.

The ability to release cell-free infectivity by knockdown of UL128 from otherwise cell-associated recent isolates can greatly facilitate such research attempts, as it becomes possible for the first time to synchronize and dose infections with freshly isolated HCMV. This allows the application of inhibitors at a chosen time point after infection and to study the effect on cell-associated spread, as the cell-free virus released after knockdown of UL128 switches back to cell-associated mode in the next round of infection. Up to now such experiments have only been possible with the tet-regulated Merlin variants, in which the UL128 region could be switched off in HFFFtet cells expressing the tet repressor [39]. This virus continues to have unique advantages in that it is available as a bacterial artificial chromosome and thus can be genetically modified, including the introduction of targeted mutations, fluorescent tags, or reporter genes. However, the finding that most of the isolates that we analyzed by quantitative immunoblotting expressed far higher levels of gO than Merlin indicated that fresh isolates may be preferable or an important adjunct for certain questions. In addition, this knockdown approach allows to transfer cell-free virus from the initial isolation culture in fibroblasts to epithelial cell culture, and thereby reduce selective pressure on the UL128 gene locus.

It was not trivial, but also not entirely unexpected, that partial knockdown of UL128 was sufficient to induce cell-free infectivity. In principle, the appearance of infectious virus in the supernatant may either be due to a complete knockdown in a subset of the transfected cells or to a partial knockdown on the level of individual cells. While we cannot differentiate these two possibilities by our immunoblotting results, the fact that epithelial cells could be infected with such supernatants argues for the latter explanation. If UL128 was completely knocked out in a subset of cells, the respective progeny would be non-endotheliotropic due to the lack of pentamer. In contrast, a partial knockdown on the single cell level would result in the release of progeny virions with reduced levels of pentamer, which are sufficient to mediate entry into endothelial and epithelial cells. A variety of laboratory strains have been described that combine extended cell tropism, due to expression of the pentamer, with highly efficient cell-free spread. All share the characteristic that the pentamer is expressed either at low levels or in a less functional state compared to the more cell-associated laboratory strain Merlin [40]. Many analyses with these laboratory strains suggested that higher pentamer levels always come at the expense of lower trimer levels, and vice versa [53,54,55]. We were, therefore, surprised to find that, in our set of clinical isolates, the levels of UL128 and gO were positively rather than negatively correlated. It would be interesting to see whether these differences in intracellular expression levels also lead to different levels of trimer and pentamer in virion particles, but this was beyond the scope of this study as too few particles can be harvested from such recent clinical isolates. We hope that further improvements regarding the release of cell free infectivity may allow appropriate analyses in the future. Regarding the question of which of the known laboratory strains can best represent clinical isolates, this immunoblot analysis showed that both strain Merlin and strain TB40/E have extremely imbalanced trimer^low^/pentamer^high^ and trimer^high^/pentamer^low^ expression patterns, and both are markedly different from recent isolates. Even in these isolates, genetic changes during the limited number of passages cannot be excluded. It is tempting to speculate that application of UL128 knockdown to primary isolation cultures may enable similar analyses at time points when genetic alterations are even less likely. It will be interesting to analyze in the future what determines the overall level of gH/gL complexes in such isolates, including a possible contribution of UL148 and US16 [55,56,57,58,59]. Of note, complete knock-out of expression from the UL128 locus by introducing dual-stop mutations into each of the UL128 locus genes only moderately increased gO levels. This indicates that gH/gL complexes, which are free due to lack of UL128 locus proteins, cannot not be quantitatively complexed by gO to form trimers. Apparently, low expression of gO itself, rather than overexpression of the UL128 locus, limits the amount of trimer that can be achieved in strain Merlin. This is consistent with recent findings with conditional repression of the pUL128 locus [29].

In conclusion, our knockout experiments with the cell-associated model virus Merlin and knockdown experiments with recent clinical isolates clearly demonstrate the role of the herpesviral fusion machinery in cell-associated spread and provide an approach to facilitate research on recent HCMV isolates.

## Figures and Tables

**Table 1:** Primers used for the introduction of stop codons into glycoprotein genes.

Primer	Sequence (5′-3′)
UL55stop_for	ggaatccaggatctggtgcctggtagtctgcgttaacttgtgaatcgtccgtctgggttaagcggtttcctcatcttctacaggatgacgacgataagtaggg
UL55stop_rev	gagtagcagaagttccacgagtagaagatgaggaaaccgcttaacccagacggacgattcacaagttaacgcagactaccacaaccaattaaccaattctgattag
UL55stop_short_for	ggaatccaggatctggtgcc
UL73stop_for	taagcatcgtggcggtggtgtgatggagtggaacacactatgattaggtcttttggtttaatcggtagtggcaagttccaaaggatgacgacgataagtaggg
UL73stop_rev	tgctagcagtcgacgtattgttggaacttgccactaccgattaaaccaaaagacctaatcatagtgtgttccactccatcacaaccaattaaccaattctgattag
UL73stop_short_for	taagcatcgtggcggtggtg
UL73stop_for M3	caacgtgatgagaccacatgctcacaatgatttttacaattgacattgtacatcgcattagtatgagctttcactgtccagaggatgacgacgataagtaggg
UL73stop_rev M3	tattccaccaggctgcaaagctggacagtgaaagctcatactaatgcgatgtacaatgtcaattgtaaaaatcattgtgagcaaccaattaaccaattctgattag
UL73stop_for_short M3	caacgtgatgagaccacatg
UL75 stop_for	cgctatgcggcccggcctccccttctacctcaccgtcttctaggtctacctccttagttgactaccttcgcaacgatatggaggatgacgacgataagtaggg
UL75 stop_rev	cttcggatgcggcgtctgcgccatatcgttgcgaaggtagtcaactaaggaggtagacctagaagacggtgaggtagaaggcaaccaattaaccaattctgattag
UL75 stop_short_for	cgctatgcggcccggcctcc
UL100stop_for	cgtggactttgaaaggctcaacatgtcggcctacaacgtatgacacctgcacacgccttaacttttcttagactcggtgcaaggatgacgacgataagtaggg
UL100stop_rev	acacggcgtagcacaccaactgcaccgagtctaagaaaagttaaggcgtgtgcaggtgtcatacgttgtaggccgacatgtcaaccaattaaccaattctgattag
UL100stop_for_short	cgtggactttgaaaggctca
UL115stop_for	ctctcatcgtgccgcagacttgatgtgccgccgcccggattgaggcttctctttctcataaggaccggtggtactgctgtgaggatgacgacgataagtaggg
UL115stop_rev	tgggcagcagaaggcaacaccacagcagtaccaccggtccttatgagaaagagaagcctcaatccgggcggcggcacatcacaaccaattaaccaattctgattag
UL115stop_short_for	ctctcatcgtgccgcagact
Kanamycin universal reverse	caaccaattaaccaattctga

Primer sites encoding stop codons are underlined.

**Table 2:** Primers used for the introduction of stop codons into genes of the UL128 locus.

Primer	Sequence (5′-3′)
UL128stop_for	acggctgagattcgcgggatcgtcaccaccatgacctagtcattgacatgacaggtcgtacacaacaaggatgacgacgataagt
UL128stop_rev	gtagttgcagctcgtcagtttgttgtgtacgacctgtcatgtcaatgactaggtcatggtggtgacgcaaccaattaaccaattctga
UL128stop_short_for	acggctgagattcgcgggat
UL130stop_for	ctgcctgcttctgtgcgcggtttgggcaacgccctgtctgtagtctccgtggtcgtaactaacagcaaaccagaatccaggatgacgacgataagt
UL130stop_rev	gtttagaccatggcggggacggattctggtttgctgttagttacgaccacggagactacagacagggcgttgcccaaacaaccaattaaccaattctga
UL130stop_short_for	ctgcctgcttctgtgcgcgg
UL131 stop_for	gtctgtttgtctgtgcgccgtggtgctgggtcagtgccagtaggaaaccgcggaataaaacgattattaccgagtaccaggatgacgacgataagt
UL131 stop_rev	agcacgcgtcccagtaatgcggtactcggtaataatcgttttattccgcggtttcctactggcactgacccagcaccacaaccaattaaccaattctga
UL131 stop_short_for	gtctgtttgtctgtgcgccg
Kanamycin universal reverse	caaccaattaaccaattctga

Primer sites encoding stop codons are underlined.

## Supplementary Materials

The following are available online at https://www.mdpi.com/article/10.3390/v13040614/s1, Figure S1: Workflow for amplification of the UL128, UL130, and UL131A open reading frames from different samples for determination of the DNA sequence, Figure S2: DNA sequences of the UL128, UL130 and UL131A open reading frames from samples before and after knockdown of UL128.
