# Supplementary material for: Role of Envelope Glycoprotein Complexes in Cell-Associated Spread of Human Cytomegalovirus
Source: Viruses. 2021 Apr 2;13(4):614. doi: 10.3390/v13040614 (PMC8066785; doi:10.3390/v13040614)
Supplement: Supplementary file 1 [file viruses-13-00614-s001.zip › suppl/210330 Weiler et al. figure S2.docx]

**Supplemental data figure S2:**

**
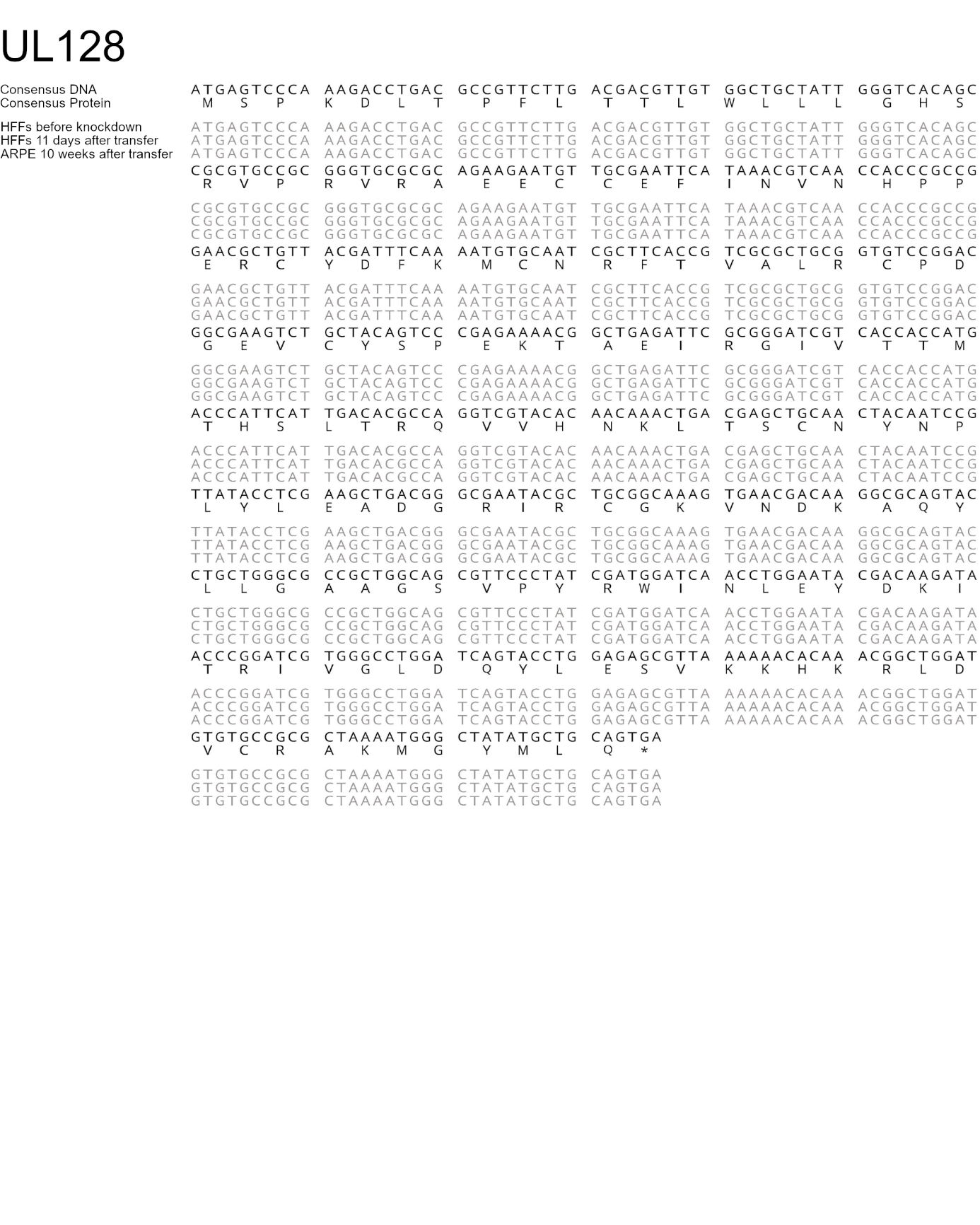
**

**
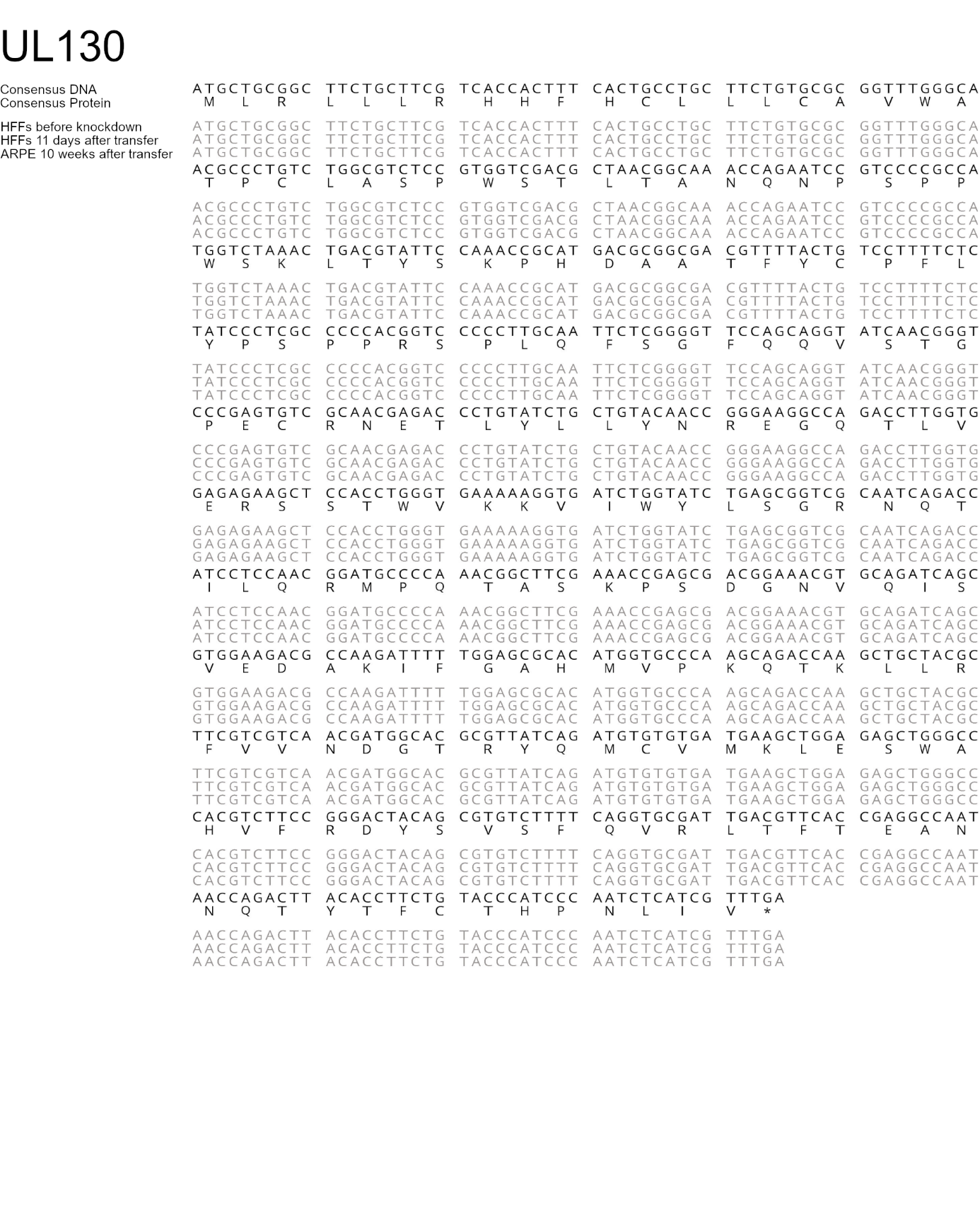
**

**
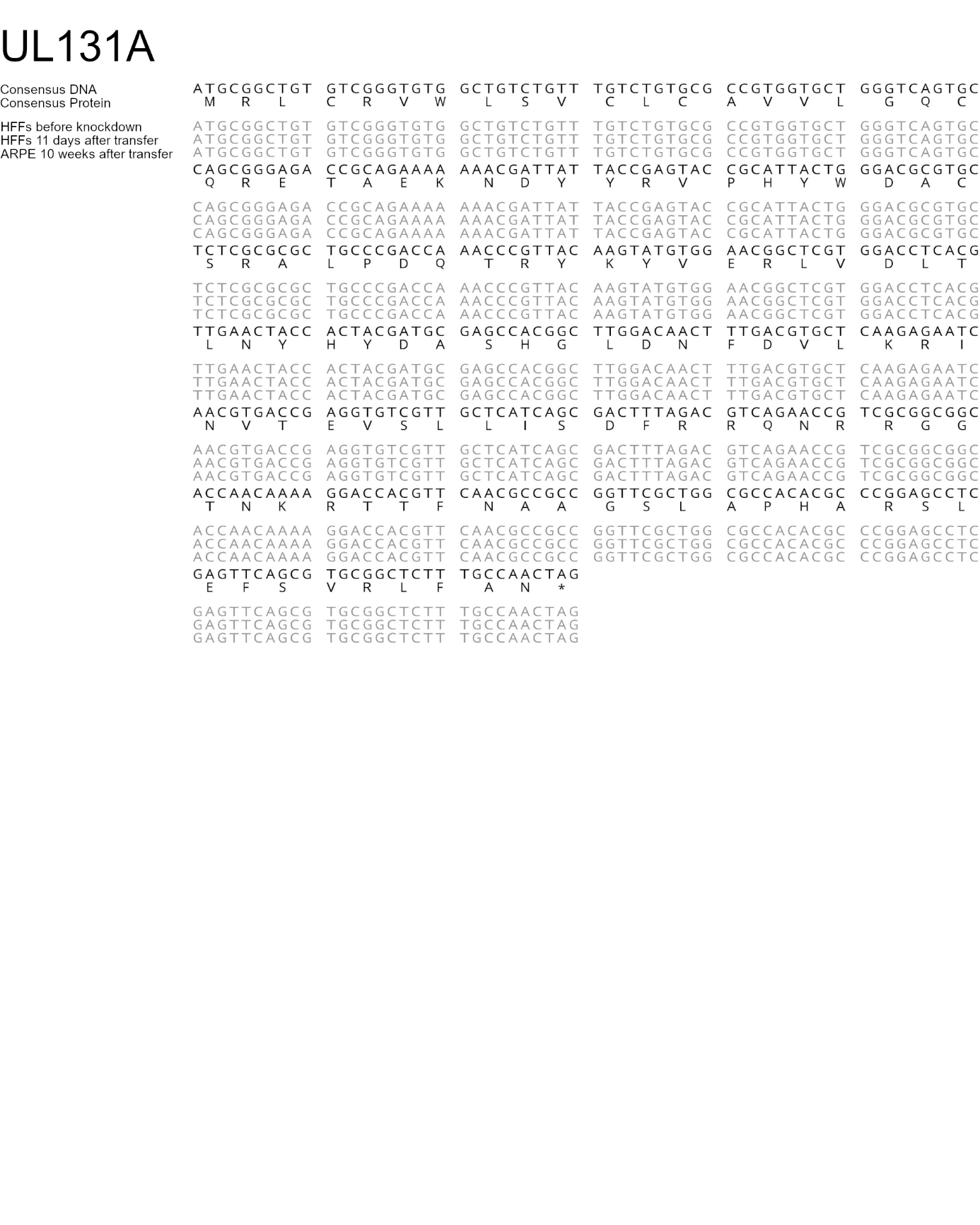
**

**S2: DNA sequences of the UL128, UL130 and UL131A open reading frames from samples before and after knockdown of UL128.** Amplification products of UL128, UL130, and UL131A obtained from samples of the isolate in HFFs before knockdown of UL128, HFFs 11 d after transfer of the released cell-free virus, and ARPE19 cells after long-term propagation of the transferred virus for 10 weeks were used to determine the respective DNA sequences. The black sequence represents the consensus sequence generated from the sequences of all three samples, along with their translation below. The three gray sequences represent the sequences obtained with the three different samples. All open reading frames appeared intact and encoded full-length proteins. No differences were found between the three samples for any of the open reading frames.
