# Supplementary material for: Role of Envelope Glycoprotein Complexes in Cell-Associated Spread of Human Cytomegalovirus
Source: Viruses. 2021 Apr 2;13(4):614. doi: 10.3390/v13040614 (PMC8066785; doi:10.3390/v13040614)
Supplement: Supplementary file 1 [file viruses-13-00614-s001.zip › suppl/210331 Weiler et al. figure S1.docx]

**Supplemental data Figure S1.**


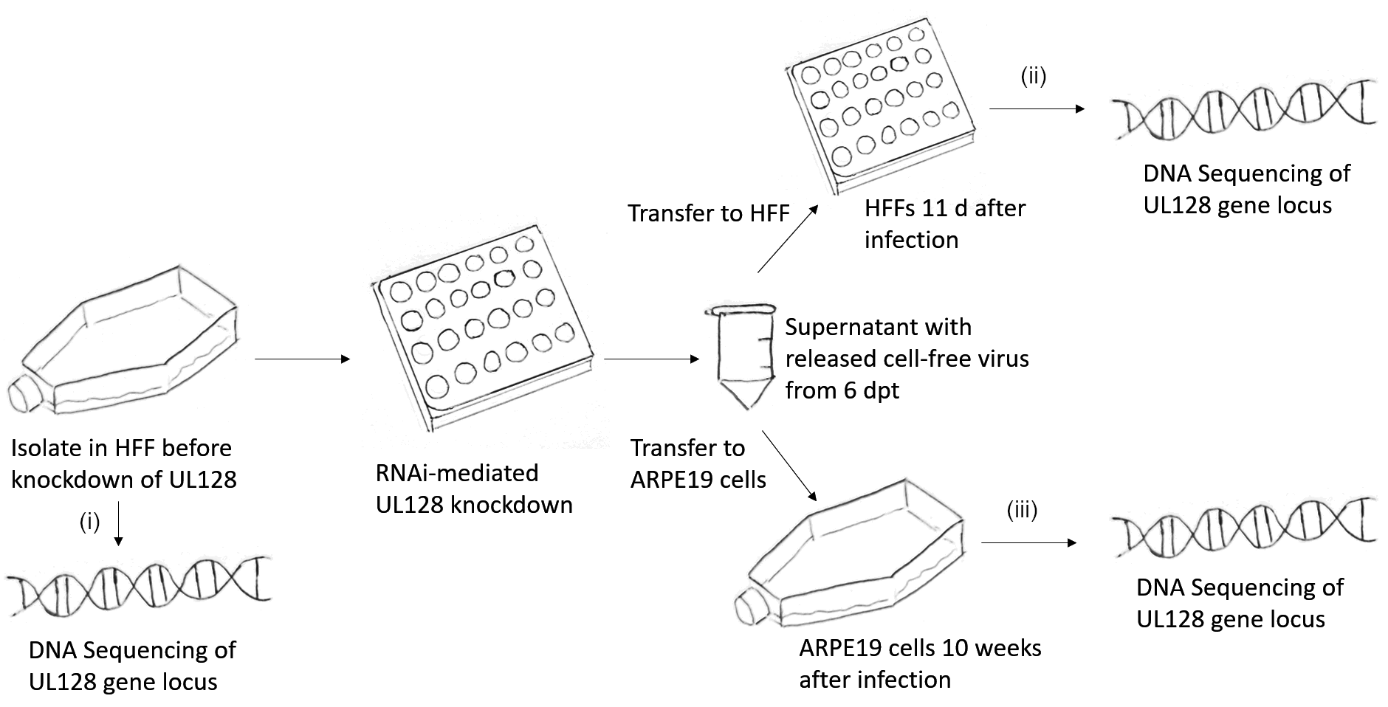


**S1: Workflow for amplification of the UL128, UL130, and UL131A open reading frames from different samples for determination of the DNA sequence.** To investigate the stability of the UL128 gene locus after siRNA-mediated knockdown, supernatants from day 6 after transfection were transferred to HFFs and ARPE-19 cells, and each of the three open reading frames UL128, UL130, and UL131A was amplified from samples of (i) the isolate in HFFs before knockdown of UL128, (ii) HFFs 11 d after transfer of the released cell-free virus, and (iii) ARPE19 cells after long-term propagation of the transferred virus for 10 weeks. The amplification products were then used to determine the DNA sequence of the UL128, UL130, and UL131A open reading frames.
